# A review of methods to measure tendon dimensions

**DOI:** 10.1186/s13018-018-1056-y

**Published:** 2019-01-14

**Authors:** Alex Hayes, Katrina Easton, Pavan Teja Devanaboyina, Jian-Ping Wu, Thomas Brett Kirk, David Lloyd

**Affiliations:** 1 Department of Mechanical Engineering, Curtin University of Technology, Perth, Western Australia Australia; 2 Medical Engineering and Physics, Royal Perth Hospital, Perth, Western Australia Australia; 3 Centre for Musculoskeletal Research, Menzies Health Institute Queensland, Griffith University, Gold Coast, Queensland Australia; 4 Academy of Advanced Interdisciplinary Studies and the Department of Biomedical Engineering of Southern University of Science and Technology, No 1088, Xueyaun Rd, Xili, Nanshan District, Shenzhen City, 518055 Guangdong Province China; 5 Faculty of Science and Engineering, Curtin University of Technology, Perth, Western Australia Australia; 6 Independent Consultant, Salt Lake City, USA

**Keywords:** Tendon, Dimensions, Measurements, In vivo, Ex vivo

## Abstract

Tendons are soft tissues of the musculoskeletal system that are designed to facilitate joint movement. Tendons exhibit a wide range of mechanical properties matched to their functions and, as a result, have been of interest to researchers for many decades. Dimensions are an important aspect of tendon properties.

Change in the dimensions of tissues is often seen as a sign of injury and degeneration, as it may suggest inflammation or general disorder of the tissue. Dimensions are also important for determining the mechanical properties and behaviours of materials, particularly the stress, strain, and elastic modulus. This makes the dimensions significant in the context of a mechanical study of degenerated tendons. Additionally, tendon dimensions are useful in planning harvesting for tendon transfer and joint reconstruction purposes.

Historically, many methods have been used in an attempt to accurately measure the dimensions of soft tissue, since improper measurement can lead to large errors in the calculated properties. These methods can be categorised as destructive (by approximation), contact, and non-contact and can be considered in terms of in vivo and ex vivo.

## Background

### Tendons

Tendons are a soft connective tissue designed to efficiently transfer loads generated by muscles to the skeletal system, facilitating joint movement [1, 2]. These can be found as rounded cords, strap-like bands, or flattened ribbons, depending on their function. Tendons exhibit a complex hierarchical structure arranged longitudinally to resist the direction of most tension [3]. This hierarchy exhibits complex micromechanics that allow the muscle-tendon-bone construct to act efficiently. Tendons demonstrate viscoelastic behaviour [4–6]; that is, they exhibit time- and strain rate-dependent properties [4, 7, 8].

Tendons primarily consist of water (65–70% wet weight) and collagen type-I (70–80% dry weight), with different types of collagen fibres, elastin, proteoglycans, and glycolipids making up the remainder [4, 9–13]. Collagen type-I represents approximately 95% of all collagen in the tendon, with the remaining 5% collagen type-III and type-V [14–17]. Collagen type-III is primarily found in aged and healing tendon, and normal tendon is mainly limited to the insertion sites of highly stressed tendons and in the endo- and epitenons [14, 16]. Glycosaminoglycans, glycoproteins, and proteoglycans make up the non-collagenous matrix components [10, 15, 16, 18]. The non-collagenous matrix plays an important role within the tendon, including contributing to the mechanical properties [19], particularly the viscoelastic behaviour [20, 21]. Tendons have a low cell density (< 5%) and this is thought to contribute to their limited healing capacity [10, 14].

Tendons exhibit properties such as stiffness, resilience, and strength, which allow for efficient transfer of forces from muscle to bone [22]. It has been observed that tendons exhibit a wide range of mechanical properties due to the breadth of functions performed [14, 23, 24]. As a result, tendon properties have been of interest to researchers for many decades [25–27]. Knowledge of the mechanical properties not only contributes to understanding of the tendon function but also provides inputs for computer simulations of the human body [28].

The Achilles tendon is generally regarded as the strongest tendon in the human body [22, 29–31]. Forces of 1–4 kN have regularly been measured during jumping and cycling, and peak forces of 9 kN, or 12.5 times body weight, have been measured during running at full speed [15, 16, 18, 30, 32]. The breaking stress of tendon is estimated to be 50–100 MPa [14, 26, 32–36]. Stresses in excess of 70 MPa have been measured in vivo [32] and are regularly reported between 30 and 60 MPa [8, 37–39]. Stress in the Achilles tendon in vivo has been estimated to be as high as 110 MPa [40]. Hence, peak stress in vivo may in some cases exceed the measured ultimate tensile strength (UTS) of the tendon [18], illustrating the complexity of tendon mechanics in vivo.

### The problem

Musculoskeletal conditions are common, with 30 million cases of injury reported annually worldwide [41]. Despite being the largest and strongest tendon in the body, the Achilles is reported to be involved in the most sports-related tendon injuries [31]. For example, injury is regularly seen in ageing athletes who participate in repetitive explosive activities [4, 42]. It has been reported that ruptured tendons show significant degeneration compared with normal controls [43, 44], suggesting disease may precede and possibly contribute to rupture. Degenerated tendons exhibit decreased mechanical properties, such as stiffness and UTS [45, 46], and are generally observed to be disordered with a larger cross-sectional area (CSA), a lower stiffness, and a lower elastic modulus [45, 47].

Disease of the tendon, known as tendinopathy, is characterised by pain and reduced mobility and functionality. The pathology is complex, including disordered healing causing fibre disruption and disorientation, generally with an absence of inflammatory cells. The aetiology and progression of the disease are not well known, leading experts to coin the term ‘tendinopathy’ to describe the clinical presentation of the condition [48, 49]. The prevalence of tendinopathy has been estimated at 11.83 per 1000 persons per year, with an incidence rate of 10.52 per 1000 persons per year [50]. Achilles tendinopathy has been reported to be as prevalent as 6–9% of some populations [51], with 4% of sufferers going on to suffer rupture of the tendon [52].

Tendinopathy is traditionally considered an overuse injury caused by repetitive strain of the tendon [4, 15, 18, 53]. While widely accepted, this view is unproven and has been challenged by several authors, including Arnoczky et al. [54] and Rees et al. [55]. Previous studies attempting to elucidate the aetiology of tendinitis found that repetitive loads caused microscopic failure of the collagen matrix, triggering an inflammatory response [4, 56]. However, the lack of inflammatory markers in many cases means tendinitis can only be confirmed with histology, and thus, tendinopathy is the preferred term [55]. A recent hypothesis proposes that microdamage may lead to isolation of segments of the tendon which in turn leads to underuse [54].

The consequences of tendon injuries are pronounced, and the underlying causes and tissue responses must be better understood in order to develop improved treatment and prevention techniques. Due to a lack of evidence-based management, treatment has traditionally been conservative, with surgery considered the last resort due to the lack of evidence for its efficacy [4, 57]. Conservative management techniques primarily aim to relieve the symptoms of tendinopathy [15]. Counterintuitively, many conservative treatment options now involve applying load to the tendon via eccentric exercise, but this remains controversial [18, 30, 55].

### Importance of measurements

Changes in the dimensions of tissues are often seen as a sign of injury and degeneration, as an increased area may suggest swelling, inflammation, and general disorder of the tissue. This is true in tendinopathy, where affected tendons have exhibited larger CSA compared to controls [47]. CSA is also an important measurement for determining the mechanical properties and behaviours of materials, particularly the stress, strength, and elastic modulus of the material. This makes CSA a significant measurement in the context of a mechanical study of degenerated tendons. Large errors in stress calculations can occur due to inaccurate measurement of an object’s CSA [58].

One assumption commonly made when assessing the mechanical properties of tendon is that the CSA remains constant and is uniform along the length of the specimen. Thus, CSAs are measured at a single location or averaged over several measurements, thereby allowing for loads to be converted to engineering stresses post-testing (*σ*_E_ = *F*/*A*_0_, where *σ*_E_ represents the engineering stress, *F* is the force, and *A*_0_ is the original area). According to Poisson’s effect, the transverse strain changes with axial strain, resulting in changes to CSA. In many situations, the engineering stress gives a reasonable approximation; however, even at low loads, a difference can be detected [59]. It is therefore desirable to measure the true stress, the force divided by the actual area (*σ*_T_ = *F*/*A*_i_), to better estimate the stress experienced by soft tissue. This requires measurement of both instantaneous and local CSA along a sample, which can be used to better define the relationship between stress and strain [59], as well as to develop more accurate geometric models for finite element analysis (FEA) [60]. The limitation is due, in part, to the lack of simple three-dimensional (3D) measurement techniques capable of measuring CSA along the length of the specimen.

Soft biological tissue samples have irregular shapes and are load-, rate-, and time-sensitive. Therefore, the measurement technique must be considered in order to achieve an accurate result. Additionally, tendon dimensions are useful in planning harvesting for tendon transfer and joint reconstruction purposes [61–63].

Over the years, many methods have been employed in an attempt to accurately measure CSA of soft tissue, since inaccuracies can result in large errors when calculating the stress [58]. These methods can be categorised as destructive (by approximation), contact, and non-contact [64]. Furthermore, these methods can be considered in terms of in vivo and ex vivo techniques.

## In vivo measurement techniques

Tendon dimensions are often used as an indication of injury and degeneration [47], as well as in the planning of tendon harvesting [61–63]. Therefore, clinically available in vivo measurement techniques are important. Modalities such as ultrasonography (US), computed tomography (CT), and magnetic resonance imaging (MRI) are prevalent in the clinical environment (Table 1). In particular, MRI and US are useful imaging modalities for visualising tendon morphology [65, 66].

**Table 1:** Summary of techniques to measure the dimensions and geometries of soft tissues and their clinical value

Technique	Papers	Advantages	Disadvantages	Clinical value
Anthropomorphic correlations	[62, 74, 75, 85]	• Simple • Fast	• Limited reliability • Inherent errors due to assumptions • Not truly patient-specific • Lack of quality physical measurements for comparison	Limited clinical usefulness due to assumptions and low reliability
Ruler	[76]	• Simple • Fast	• Unable to measure two- or three-dimensional geometry	Clinically useful due to simplicity
Computed tomography (CT)	[63, 67, 135, 136]	• Readily available diagnostic imaging technique • Non-contact • Non-invasive • Three-dimensional	• Poor discrimination of soft tissues • Radiation dose • Lack of quality physical measurements for comparison	Limited clinical usefulness due to poor discrimination of soft tissues
Computed tomography with contrast	[68, 69]	• Improved differentiation of soft tissues • Non-contact • Non-invasive • Three-dimensional	• Limited to ex vivo evaluations • Potential deformation of tissue • Poor discrimination of soft tissues • Radiation dose	Limited clinical usefulness due to difficulty applying contrast agents
Magnetic resonance imaging (MRI)	[61, 62, 70–84, 137, 138]	• Readily available diagnostic imaging technique • Able to differentiate soft tissues • Image quality can be improved with digital post-processing • Safe • Non-contact • Non-invasive • Three-dimensional	• Expensive • Slow • Lack of robust methodologies • Conflicted reports of accuracy and reliability • May not adequately resolve paratenon • Lack of quality physical measurements for comparison	Clinically useful due to clear differentiation of soft tissues
Ultrasound, 2D (2DUS)	[39, 45, 66, 73, 78, 79, 85–115]	• Readily available for diagnostic imaging technique • Safe • Fast • Non-invasive • Inexpensive • Able to differentiate soft tissues	• Two-dimensional • Conflicted reports of accuracy and reliability for deep tendons • Results dependent on operator, pressure, position, and orientation • Lack of quality physical measurements for comparison • Unable to detect paratenon • Requires contact	Clinically useful for superficial tendons
Ultrasound, 3D (3DUS)	[117–121]	• Accurate • Reliable • Repeatable • Reduced operator-, position-, and orientation-dependency • Safe • Fast • Non-invasive • Three-dimensional • Able to differentiate soft tissues	• Unable to detect paratenon • Requires contact • Pressure dependency	High clinical usefulness for superficial tendons
Sectioning	[27, 124, 125]	• Accurate • Repeatable • Can be reconstructed to three-dimensional	• Destructive	Low clinical usefulness due to destructive nature
By estimation	[126]	• Simple • Fast	• Inherent errors due to shape assumptions • Does not capture geometry • Affected by measurement technique (e.g. ruler)	Clinically useful for comparative measurements
Area micrometry	[126–128]	• Simple • Fast • Repeatable	• Underestimates area • Contact • Does not capture geometry	Clinically useful for comparative measurements
Casting	[129–131]	• Accurate • Reliable • Repeatable • Three-dimensional • Ability to revisit measurements	• Slow • Contact • Requires tissue to be isolated • Unable to visualise internal structures	Clinically useful for some tissues, particularly resected tissues
Shadow amplitude	[126]	• Accurate • Non-contact	• Poor repeatability • Unable to visualise internal structures	Limited clinical usefulness
Laser micrometry	[4, 59, 60, 64, 132, 134, 139, 140]	• Fast • High accuracy • Repeatable • Reliable • Non-contact • Two-dimensional	• Affected by specimen geometry, concavities, opacity, reflectivity, and orientation • Unable to visualise internal structures	Limited to external and ex vivo measurements
Laser scanning	[142]	• Fast • High accuracy • Repeatable • Reliable • Non-contact • Three-dimensional	• Affected by specimen geometry, concavities, surface refraction, alignment of sample, opacity, reflectivity, and orientation • Limited viewing window for three-dimensional reconstruction • Unable to visualise internal structures	Clinically useful for 3D surface measurements. Limited to external and ex vivo tissues
Photogrammetry	[143]	• High accuracy • Repeatable • Reliable • Non-contact • Three-dimensional • Photorealistic reconstruction	• Affected by concavities • Unable to visualise internal structures	Clinically useful for 3D surface measurements. Limited to external and ex vivo tissues
Structured white light (SWL)	[144–146]	• Fast • High accuracy • Repeatable • Reliable • Non-contact • Three-dimensional • Photorealistic reconstruction	• Affected by small concavities • Unable to visualise internal structures	Clinically useful for 3D surface measurements. Limited to external and ex vivo tissues
Digital image correlation (DIC)	[147–155]	• Fast • High accuracy • Repeatable • Reliable • Non-contact • Three-dimensional	• Requires sample preparation • Affected by small concavities • Unable to visualise internal structures	Clinically useful for 3D surface and strain measurements. Limited to external and ex vivo tissues

### Computed tomography

CT is a common imaging technique for diagnostic and preoperative planning, making it an attractive option for observing tendon dimensions. However, the low x-ray attenuation of soft tissues can make them difficult to distinguish. Yasumoto et al. [63] demonstrated that CT can be an effective tool for measuring the length, but not the area, of the semitendinosus for anterior cruciate ligament (ACL) graft planning, while Schepull et al. [67] utilised CT to measure the area of the Achilles tendon. This is likely due to the difference in volume of soft tissue surrounding each tendon, resulting in easier differentiation of the Achilles tendon compared to the semitendinosus. One method of improving the contrast of the tissues is to utilise a staining agent [68, 69]. While improving the quality and information available in the acquired images, this technique is limited to ex vivo evaluations and prolonged staining of the tissue has been associated with shrinkage and deformation of the tissue [68]. Due to the diffusion times required for the agents, it is unlikely that this technique would be transferable to a clinical setting.

### Magnetic resonance imaging

MRI has been used to measure Achilles tendon [70–72] and patellar tendons [73–76]. Despite MRI being a common technique, few studies have reported robust methodologies for measuring tendon dimensions. Many reported techniques have focused on finding a correlation between anthropomorphic measurements, particularly height, and the dimensions of graft tendons for preoperative planning [62, 74, 75]. Similarly, CSA measurements of tendon graft using MRI have been shown to correlate with final graft CSA in ACL reconstruction [61, 62, 77–81]. Tendon-only length was also shown to correlate with the intraoperative tendon-only length of gracilis and semitendinosus tendons [82], while no correlation has been reported between preoperative diameter measurements and the final diameter [80]. The majority of studies calculated the CSA using a graft sizing block and ‘by estimation’ approach, thereby limiting the accuracy of the intraoperative tendon measurements.

Chang et al. [76] performed intraoperative patellar tendon measurements using a ruler, showing high accuracy and reliability of the measurements. Hamada et al. [83] reported a close positive correlation between preoperative MRI and intraoperative CSA measurements using an area micrometre. However, the MRI measurements were shown to underestimate the intraoperative measurements by almost 10%. This was also seen by Couppe et al. [84], in a horse cadaver model, when comparing measurements on greyscale MRI to ex vivo casting measurements. An improvement in accuracy was demonstrated by adjusting the colour scale of the MRI images to better delineate the tendon from the surrounding tissues [84]. In defining the tendon boundary, consideration must be given to the thickness of the paratenon [72], the boundary of which may not be clear on greyscale imaging.

### Ultrasonography

US is an inexpensive, safe, fast, and reliable non-invasive technique for imaging tendon, including pathology and geometry [39, 45, 85–89]. US has been shown to be a suitable alternative to MRI for tendon measurements [79] and is a less expensive technique [90]. US has been used to measure tendon dimensions, including thickness, length, and area, and is particularly suited to large superficial tendons such as the Achilles [91–104] and patellar tendons [39, 45, 89, 105, 106].

Similar to MRI, US has been used to look at correlations in tendon size with anthropomorphic measurements. Patel and Labib [85] investigated various Achilles tendon parameters (length, width, thickness, and CSA) and found they correlated positively with subject height, weight, tibia length, and foot size. The Achilles tendon CSA correlated significantly with the activity level.

The results of the accuracy and reliability of US in literature has been conflicted. Some reports have described US as unreliable as a technique for measuring tendon in vivo, while others describe it as reliable and accurate [85, 107, 108]. For example, US measurements of the gracilis and semitendinosus have been shown to be both correlated [78, 79] and not correlated [109] with preoperative calculations for hamstring graft in anterior cruciate repairs. Operator dependence is a known factor in US [66, 90] and is a known limitation in the accuracy and reliability of the technique [86], while position and measurement location are highly important for reliability [110].

Authors such as Ying et al. [96] and Barfod et al. [111] have proposed clinically applicable, standardised methods for measuring tendon geometry in an attempt to improve the reliability and accuracy of US measurements independent of the operator. A work by Skou and Aalkjaer [86] found that changes in a patellar tendon larger than 0.7 mm could be detected by the same operator, while changes of 1 mm could be detected by different operators. They highlighted the importance of standardised methods in improving US measurements. A recent review of papers analysing diagnostic measures of tendon size reported that the measurement error associated with reliability is less than the difference in the size of symptomatic and asymptomatic tendons [108]. Recent studies, using MRI for baseline measurements, demonstrated the accuracy and reliability of US for measuring tendon length in the Achilles tendon. Reeves et al. [88] measured CSA of the patellar tendon using US, finding strong agreement in measurements taken by the same operator on separate days. A follow-up study by Reeves et al. [87] showed a close inter-method agreement with MRI and included a morphometric analysis of a phantom using MRI to determine accuracy.

In contrast, Ekizos et al. [73] and Bohm et al. [112] found US unsuitable for accurate measurement in vivo. Bohm et al. [112] and Kruse et al. [110] reported that US underestimated the CSA of the Achilles tendon, while Ekizos et al. [73] found US had a low reliability, including time, position, and observer differences. Some of the limitations included low visibility and blurry boundaries in the US images. Transducer pressure has also been shown to affect tendon measurement [91, 95, 110, 113].

A significant shortcoming of many studies is the lack of high-quality physical measurements of the tendons. Kruse et al. [110] noted that, without physical measurements, it is possible that MRI overestimates the measurement, rather than the reported conclusion that US underestimates tendon CSA.

The accuracy of US has been demonstrated in ex vivo testing. Noguchi et al. [114] demonstrated US measurements to be as effective in measuring tendon and ligament specimens as ‘by estimation’ methods, using digital callipers, while preserving the morphology of the tissue. A significant limitation of the study was the assumption of a rectangular tendon for the purposes of estimating the CSA. A second limitation is the risk of overestimating the CSA due to fluid absorption, as the tissue must be imaged in a bath of saline which may affect the physiological hydration of the tissue. Du et al. [115] used US and laser micrometry in a customised rig to measure tendon dimensions during mechanical loading. US measurements of thickness were found to correlate highly with the laser measurements with increasing load. No measurement of CSA using US was reported for comparison.

Three-dimensional freehand ultrasound (3DUS) uses a combination of two-dimensional US (2DUS) and 3D motion capture to generate a 3D reconstruction of tissues. This technique has been shown to be accurate and reliable against MRI and phantoms [116, 117], and may overcome several limitations of 2DUS, including probe position and orientation. 3DUS has been used primarily on the Achilles tendon [116–122], with high reliability [118, 121] and repeatability [116].

Fan [122] demonstrated freehand technique, using a motion sensor, to create a 3D reconstruction of the Achilles tendon. This technique utilised only one subject, with no assessment of reliability or accuracy. Obst et al. [117] demonstrated the accuracy of the technique using phantoms as well as the reliability of in vivo measures of the Achilles tendon volume, length, and average CSA. A limitation of this technique is the inability to detect the thickness of the tendon paratenon, epitenon, and peritendinous space [118], which has previously been identified as a source of measurement error [72].

### Limitations

While there are various ways to measure tendon in vivo, the choice of measurement technique must be evaluated for its suitability to the experiment. The importance of accurate measurement has been well described [58]. In addition to improved calculation of tendon stresses and strains, knowledge of tendon dimensions has been shown to aid in planning involving tendon grafting [61, 62, 76–83] and in the identification of tendon disorders [47].

The lack of consensus in the literature is a confounding factor in deciding which technique to use. For example, it has been reported that tendon dimensions differ between US and MRI [110, 113] and are, therefore, not interchangeable [110] except under certain conditions [123]. It has also been reported that both techniques are independently reliable [110] and, conversely, that the reproducibility of MRI and US is a limitation [113]. These conflicting findings can make it difficult to determine which technique is most suitable for a particular study. A limitation of many of these studies is the lack of physical measurements for comparison [123].

This limitation is a recurring theme in the literature. The lack of high-quality control measurements when assessing the accuracy of in vivo measurement techniques makes it difficult to adequately compare between studies and between methods. This limitation is in part due to the inherent difficulty in acquiring physical measurements in vivo. A significant opportunity to acquire these physical measurements is in the form of intraoperative measurements. However, these measurements tend to be performed using graft sizing blocks and rulers, which do not provide sufficient accuracy to determine the ‘true’ dimensions of the tendon. Studies by Hamada et al. [83] and Couppe et al. [84] demonstrated the importance of these ‘true’ measurements in determining the accuracy of in vivo measurement techniques.

Many advanced measurement techniques are incompatible with the in vivo environment. However, these may be useful in providing control measurements of phantom or intraoperative measurements.

## Ex vivo measurement techniques

### Contact methods

Historically, specimens were sectioned and traced, and the areas are measured using a planimeter [27]. This method is inherently destructive and prevents further testing of the sample. Chatzistergos et al. [124] measured tendon CSA by taking sections of the tissue post-rupture. This assumes no plastic deformation has occurred during testing, as this may influence the final shape of the tissue. More recently, Iriuchishima et al. [125] evaluated CSA of ACL versus the grafts used to replace them. The authors revisited Cronkite’s technique and sectioned ACL at the bone attachments and through the midsection. With the use of digital photography and image processing they were able to measure CSA with a higher degree of accuracy and reproducibility. The authors did not discuss a method for measuring CSA of the grafts in a non-destructive fashion, limiting the clinical value of the study. It was, however, noted that a 3D measurement system would provide a higher degree of anatomical accuracy due to the natural path of the ligament.

‘By estimation’ techniques have ranged from estimating the shape of the specimens and measuring the height and width of the sample to ‘fit’ the shape, to the gravimetric method of calculating the area based on the length and volume and even ocular micrometry [126]. These techniques often assume uniformity within the tissue and, while simple, can introduce errors such as those discussed by Seitz et al. [58].

Area micrometry allows for irregularity in the shape of soft tissue by using adjustable blocks to compress the tissue into a channel with known size, from which the volume can be calculated [126–128]; however, these measurements generally underestimate CSA [59].

Race and Amis [129] approached the measurement of CSA differently, taking a silicone rubber cast and creating poly-methyl methacrylate replicas of the tissue for analysis. This technique was developed further by Goodship and Birch [130] and Schmidt and Ledoux [131] using new materials and improved techniques. Images were taken of the replicas and then analysed in silico to measure the area. These newer techniques were shown to improve measurement accuracy compared to existing methods. The casting method offers the advantage of being able to revisit measurements as the cast can be preserved even after destructive testing of the tendon.

### Non-contact methods

Non-contact methods of measuring tendon dimensions offer significant advantages in terms of speed and usability. Shadow amplitude was developed in the 1960s to measure whole tissues. Of the aforementioned techniques, it was identified as the only non-destructive method able to be adapted to measure local CSAs [126]. It was also noted that there was an inherent need for refinement in the measuring of CSAs, due to the poor repeatability of the technique and inability to identify concavities.

Technological changes have led to improved non-contact devices, such as laser micrometres developed by Lee and Woo [4, 132], video dimension analysers [133], and charge-coupled device (CCD) laser sensors [134], as well as advances in medical imaging, including CT [69, 135, 136], MRI [137, 138], and US [114]. When evaluating their new technique, Race and Amis [129] pointed out that laser micrometry is potentially the most precise method of measuring the tendon; however, it is affected by specimen geometry and concavities, which also make it potentially the least accurate when dealing with complex shapes, leading researchers to develop new ways to measure CSA as technology improved.

Langelier et al. [60] developed a new computer-controlled laser micrometre based on the work of Lee and Woo [132]. The system utilised a 10-μm laser and was found to be accurate and highly repeatable, but unable to identify concavities in the tissue. Liu et al. [139] proposed the use of a coordinate measurement machine, utilising laser micrometry of 1 μm to scan the tendon. This method was shown to be more accurate than that developed by Langelier et al. [60] when scanning a standard block (0.4%, with 1.6% repeatability); however, the tendon measurements were only compared to the less accurate shape-fitting technique.

Translucency of tendon is a known issue in microscopic investigation of tissue and may inhibit laser-based measurement due to refraction of light at the surface. Langelier et al. [60] discussed this issue and attributed it to the density of the sample being insufficient to interrupt the laser beam; however, this does not exclude the issue of refraction playing a part. Neither Langelier et al. [60] nor Liu et al. [139] evaluated their techniques against reliable existing methods; thus, the accuracy and repeatability of their experiments may be lower than reported when applied to hydrated soft tissue.

Moon et al. [134] trialled CCD laser sensors to address some of these issues, finding that the new system was able to measure concavities in an accurate and repeatable fashion. It was, however, susceptible to underestimation of CSA due to laser penetration of the semi-transparent surface of the tissue. Therefore, the tendon was stained with Indian ink to provide a reflective coating. The system was also limited to objects with CSA larger than 20 mm^2^. It was noted that the improvements over other measurement techniques were negligible for rabbit ligaments and tendon [134].

Salisbury et al. [140] sought to develop a new method for characterising CSA, using a laser-slice method. The technique was effective at measuring the concavities in CSA profile and did not require any surface modification. The tendon was required to be rotated almost perfectly vertical in order to accurately measure CSA, which limited the potential use on tissues which have a 3D anatomical path. The accuracy was comparable to other methods but was deemed cheaper and more reliable when dealing with cavities than other methods.

The importance of understanding the local variations in shape and area in soft tissues has previously been identified in relation to the development of CSA measurement systems [60, 141]. These papers discussed calculating local stresses and strains based on the local shape data. This information improves understanding of how the tissue changes with load and may be used to create more accurate FEA models. To date, almost all methods have required researchers to measure CSA outside of a materials testing system (MTS). This can often mean the condition of the sample can change between the measurement of CSA and the final testing procedure [59]. It is not necessarily practical to measure the tendon immediately prior to testing, such as when using cryo-grips [59]. Therefore, in an ex vivo setting, a measurement system capable of integration with an MTS is desirable. The importance of measuring the instantaneous CSA so that true stress can be calculated has previously been discussed [59].

Pokhai et al. [59] developed a laser reflectance system for an MTS; however, it is sensitive to opacity, reflectivity, and orientation, as well as small specimen size and small concavities. Vergari et al. [64] developed a linear scanner to measure CSA of the tendon. While this method is much faster (under 2 s per measurement) than existing techniques, and also highly accurate (less than 2% error), it is limited in the shapes that are measurable, due to the linearity of the measurements. As with previous techniques, it is only able to measure one region at a time, meaning that whole tendon shape data are not available during mechanical testing. Heuer et al. [142] developed a 2D laser scanner to measure the deformation of an intervertebral disc in three dimensions. This scanner cannot distinguish tissue morphological complexities such as concavities and has a relatively limited viewing window.

Recent developments in 3D laser and structured light scanning (SLS), as well as advanced digital image correlation (DIC), have made these techniques affordable and suitable options for research.

Hashemi et al. [143] utilised a commercially available 3D photographic scanner to scan the ACL. The scanning process was approximately 30 min, from which a 3D model was generated. The accuracy was similar to that seen with early laser micrometres; however, it also lacked the ability to detect concavities. The advantage of this technique over the laser-based systems is that CSA can be calculated at any point along the length of the tissue.

Three-dimensional structured white light (SWL) scanners have been used by Nebel [144] to create photorealistic 3D models of human bodies with an accuracy of 1 mm. These models were then converted to FEA-compatible models. More recently, Ahn et al. [145] used a 3D SWL scanner to evaluate the changes in the dentoalveolar protrusion in patients before and after orthodontic work. This involved scanning the face from three angles simultaneously and reconstructing the model to ensure that any change between angles was not a product of patient movement or positioning.

Hayes et al. [146] reported a technique, using SLS, to measure CSA of biological tissue. The technique was shown to be fast, simple, and accurate, with minimal sample preparation. The technique demonstrated a high degree of repeatability and was able to capture the entire geometry, thereby enabling true stresses to be calculated along the sample.

DIC has been used to investigate the deformation of biological materials under load [147–153]. The principle of DIC is to detect gradient differences in a greyscale image to find patterns which can subsequently be tracked between images. This often requires application of an irregular pattern of similar-sized dots to the material. This has led to DIC sometimes being referred to as ‘speckle imaging’. There are currently methods of calculation able to determine sub-pixel resolution of the strain fields [154]. Commercial packages are available that utilise stereophotogrammetry to map the strain field in 3D. The limitation of this system is usually in applying the speckle pattern, which must be fine and irregular, but with good contrast to enable visualisation of the gradient. The technique allows for the use of high resolution or high-speed cameras to maximise the quality of data captured. However, a big advantage of DIC over other modalities is the ability to record local strain in addition to calculating the shape data. Evans et al. [155] have previously discussed the advantages of using DIC in mechanical testing, as it provides more information, such as differences between regions of the test sample, which would otherwise be unmeasurable.

## Conclusion

Knowledge of tendon dimensions has been shown to aid in the identification of tendon disorders and in planning for tendon grafting. Accurate measurement of tendon dimensions is important for calculating tendon stress and strain, as well as determining the difference between normal and degenerated tendon. Additionally, reliability, repeatability, and reproducibility are important considerations in the selection of measurement techniques, as they affect the ability to compare results within and between studies. Understanding these factors, as well as the environment of operation, is a key to determining the suitability of a measurement technique.

A recurring theme in the study of in vivo tendon measurements is the lack of control measurements to confirm the accuracy of the described techniques, thereby limiting the ability to compare between techniques and studies. Consensus on the results of in vivo techniques in the literature is limited and is affected by the lack of control measurements to determine the accuracy of these techniques. This severely limits the ability to compare between techniques and studies. Therefore, the use of control measurements is recommended to improve the reporting of tendon dimensions in literature.

## Abbreviations

2D: Two-dimensional
2DUS: Two-dimensional ultrasonography
3D: Three-dimensional
3DUS: Three-dimensional ultrasonography
ACL: Anterior cruciate ligament
CCD: Charge-coupled device
CSA: Cross-sectional area
CT: Computed tomography
DIC: Digital image correlation
FEA: Finite element analysis
MRI: Magnetic resonance imaging
MTS: Materials testing system
SLS: Structured light scanning
SWL: Structured white light
US: Ultrasonography
UTS: Ultimate tensile strength
